# RNA Binding Suppresses Tsg101 Recognition of Ub-Modified Gag and Facilitates Recruitment to the Plasma Membrane

**DOI:** 10.3390/v12040447

**Published:** 2020-04-15

**Authors:** Susan M. Watanabe, Madeleine Strickland, Nico Tjandra, Carol A. Carter

**Affiliations:** 1 Department of Microbiology & Immunology, Renaissance School of Medicine, Stony Brook University, Stony Brook, NY 11794-5222, USA; Susan.Watanabe@stonybrook.edu; 2 Biochemistry and Biophysics Center, National Heart, Lung, and Blood Institute, National Institutes of Health, Bethesda, MD 20892, USA; maddy.davison@NIH.gov

**Keywords:** HIV, Gag, Tsg101, ESCRT, ubiquitin, RNA, nucleocapsid

## Abstract

The ESCRT-I factor Tsg101 is essential for sorting endocytic cargo and is exploited by viral pathogens to facilitate egress from cells. Both the nucleocapsid (NC) domain and p6 domain in HIV-1 Gag contribute to recruitment of the protein. However, the role of NC is unclear when the P(S/T)AP motif in p6 is intact, as the motif recruits Tsg101 directly. The zinc fingers in NC bind RNA and membrane and are critical for budding. Tsg101 can substitute for the distal ZnF (ZnF2) and rescue budding of a mutant made defective by deletion of this element. Here, we report that the ubiquitin (Ub) E2 variant (UEV) domain in Tsg101 binds tRNA in vitro. We confirmed that Tsg101 can substitute for ZnF2 when provided at the viral assembly site as a chimeric Gag-Tsg101 protein (Gag-ΔZnF2-Tsg101) and rescue budding. The UEV was not required in this context; however, mutation of the RNA binding determinants in UEV prevented Tsg101 recruitment from the cell interior when Gag and Tsg101 were co-expressed. The same Tsg101 mutations increased recognition of Gag-Ub, suggesting that tRNA and Ub compete for binding sites. This study identifies a novel Tsg101 binding partner that may contribute to its function in recognition of Ub-modified cargo.

## 1. Introduction

The structural precursor polyprotein, Gag, encoded in the genome of HIV-1 possesses redundant “late” (L) domains required for efficient budding from the plasma membrane. The Pro-rich motif in the p6 domain of Gag (PTAP) that can fit into a P(T/S)AP-binding pocket in Tsg101 is considered the primary L domain responsible for driving budding. To date, this motif is conserved in all HIV variants isolated (Los Alamos National Laboratory (LANL) HIV database). A so-called secondary L domain (LYPX_n_L) also exists in the p6 region, so designated because it rescues budding to a degree that is cell-type dependent when Gag-P(T/S)AP-Tsg101 binding is disrupted. This motif binds the cellular ESCRT adaptor ALIX through its V domain. The Bro domain in ALIX associates with RNA that is bound to the NC domain in Gag [1] and also with the ESCRT-III factor, CHMP4 [2,3,4,5,6,7,8,9,10]. Tsg101 binds only p6 [11,12,13]. However, mutations of basic residues throughout NC or in zinc fingers (ZnFs) cause a significant decline in release of particles carrying the intact PTAP motif indicating that, in the context of NC mutants, the PTAP motif is not sufficient to drive virus release [8,9,14,15]. These findings collectively indicated that NC and the PTAP motif play cooperative roles in viral egress. Studies with NC-spacer 2-p6 (NCp15), the C-terminal region of Gag, suggest that p6 folds back and interacts with the basic ZnFs [16]. Four residues in NC, distributed throughout the two ZnFs, showed chemical shift differences in NCp9 compared to NCp15. Chamontin et al. went on to show that zinc finger 2 (ZnF2) is the element in NCp15 which, in the presence of RNA, determines whether or not Tsg101 is recognized [17]. This group further demonstrated that Tsg101, when substituted for ZnF2, rescued the budding defect caused by ZnF2 deletion. By employing a combination of the fluorescence resonance energy transfer (FLIM-FRET) technique and NMR, El Meshri et al. subsequently showed that deletion of the entire NC domain, or even just the two zinc finger motifs within this domain, alters the Gag-directed cellular distribution of Tsg101 and negatively impacts their interaction [15].

Previous studies have demonstrated that ESCRT-I can support release of the virus-like particles (VLPs) assembled by HIV-1 Gag, even when recruited to assembly sites via non-native interactions. For example, mutated retroviral Gag proteins that would otherwise remain in the cell can bud when fused directly to various proteins or domains that can recruit ESCRT-I, including ubiquitin (Ub) [18,19,20], Vps28 [21], Hrs [22], and Tsg101 [23,24]. The rendition of such rescue demonstrated by Chamontin et al. is of particular interest because (i), the deleted element, ZnF2, is not a recognized L domain and its specific contribution to budding is unclear; (ii), the p6 domain in the mutant was intact and (iii), despite the presence of an intact P(T/S)AP-bearing p6 region, rescue was achieved by providing Tsg101 itself. Thus, the Chamontin adaptation suggested that a function embodied in Tsg101 was related to the NC contribution. As they observed reduced Tsg101 accumulation at the Gag assembly site, Chamontin et al. hypothesized that ZnF_2_ deletion impairs recruitment of Tsg101. The proximal Zn finger (ZnF_1_) appears to be more critical than ZnF_2_ for the nucleic acid interactions and chaperone activity of NC and for gRNA packaging [25,26]. Deletion of ZnF_1_ does not have a major impact on budding as reflected by ELISA or Western analysis [27,28,29]. In contrast, deletion of ZnF_2_ has a greater effect on budding ([17,30]; reviewed in [31]).

The ΔZnF_2_ mutation arrests budding at an early (pre-neck formation) stage [17], as does disruption of the Tsg101 Ub binding site in the Tsg101 UEV domain [32]. Previously, we observed that co-immunoprecipitation of NCp15 from COS-1 cell lysates by anti-Tsg101 antibodies was RNase-sensitive (VerPlank 2001, PhD Dissertation). The identification of ZnF_2_ as the element critical for Tsg101 recognition, as noted above, was made using lysates from cells co-transfected with non-coding HIV-1 gRNA and GST-tagged NCp15 [17]. These observations, combined with the fact that small molecules capable of binding the Tsg101 UEV domain resemble adenine or guanine [32,33], prompted us to investigate the possibility that the UEV domain might recognize RNA. Here, we identify tRNA as a new Tsg101 binding partner that influences Tsg101 recruitment in conjunction with Ub-binding and P(T/S)AP motif recognition.

## 2. Materials and Methods

### 2.1. Production of Isolated Tsg101 UEV Domain

^15^N-labeled N-terminally His_6_-tagged Tsg101 UEV domain (residues 2-145) and ^15^N-Ub^S57C^ were expressed and purified as previously described [32]. NMR samples contained ~0.1 mM Tsg101 UEV, 20 mM potassium phosphate (pH 5.8), 50 mM NaCl, and 7% ^2^H_2_O.

### 2.2. NMR Spectroscopy (Binding Studies)

For binding studies using NMR spectroscopy, yeast tRNA (Ambion yeast tRNA, 10 mg/mL) was added to ^15^N-Tsg101 UEV or ^15^N-Ub^S57C^ (control) at a concentration of 1:1 ratio. Final buffer exchange was achieved by washing with Amicon Ultra centrifugal filters (MWCO 10 kDa for Tsg101 UEV and 3 kDa for Ub, respectively). NMR data were acquired at 300 K on a Bruker 800 MHz spectrometer, equipped with a cryogenic probe. Spectra were processed using NMRPipe [34] and analyzed using CCPN Analysis 2.4.1 [35]. Assignments were completed using standard triple-resonance experiments, as described previously [32]. ^1^H/^15^N-HSQC experiments were used to assay for binding while chemical shift perturbations were measured using ^1^H/^15^N-HSQC-TROSY experiments [36] owing to the large size of the tRNA/Tsg101 complex. Chemical shift perturbations of ^15^N-Tsg101 UEV in complex with tRNA were calculated according to the following equation: √[(H_complex_ − H_free_)^2^ + (α(N_complex_ − N_free_))^2^], where α = 0.13 = (H_max_ − H_min_) / (N_max_ − N_min_) using backbone amide ^1^H and ^15^N chemical shifts [37].

### 2.3. Plasmids and Reagents

The plasmid encoding HIV-1 Gag C-terminally tagged with green fluorescent protein (pCMV-Gag-EGFP) and the plasmid encoding full-length human Tsg101 N-terminally tagged with myc (pLLEXP1-hTSG101-myc) were previously described [32]. Using site-directed mutagenesis, pCMV-Gag-EGFP was used as a template to generate a plasmid (GagΔZnF2) expressing HIV-1 Gag lacking the ZnF2 region (ZnF2 sequence deleted: CWKCGKEGHQMKDC). The GagΔZnF2 - Tsg101 chimera was constructed using PCR to add the full length hTsg101 reading frame to the 3′ end of GagΔZnF2. Deletion mutants of the GagΔZnF2- Tsg101 chimera were constructed by substituting stop codons at Tsg101 sequences encoding amino acid numbers 140, 257, and 313. The GagΔZnF2-ΔUEV Tsg101 chimera was constructed using PCR to stitch together the reading frames for GagΔZnF2 and hTsg101′s sequence starting at the codon for amino acid 140 and ending at the stop codon. The yeast two hybrid pGBT p6 plasmid was constructed by subcloning HIV-1 Gag p6; ubiquitin sequence was added to the 3′ end of HIV-1 Gag p6 to construct pGBT p6-Ub. pGAD-hTsg101 was constructed by subcloning the Tsg101 sequences from pLLEXP hTSG101-myc.

### 2.4. Transfection and Assays for Particle Budding

293T cells (ATCC CRL-3216) were transfected with plasmids as indicated in the figure legends using Roche X-tremeGene transfection reagent (Sigma-Aldrich, St. Louis, MO, USA). At 24 h post-transfection, media was filtered (0.45 micron), and pelleted through a 20% sucrose cushion (20,000× g, 90 min). Pellets (virus-like particles) were resuspended in sample buffer and examined by Western blotting. For cell-associated Gag analysis, cell pellets were lysed (50 mM Tris, pH 7.4, 137 mM NaCl, 1.5 mM MgCl_2_, 1 mM EDTA, 1% Triton X-100, Roche cOmplete mini protease inhibitor cocktail) and centrifuged at 1000× *g* for 15 min. Supernatants were analyzed by Western blotting. Primary antibodies were: Rb anti-CA [38], anti-actin (Sigma-Aldrich, MO, USA, A4700, 1:1000), mouse anti-myc (Santa Cruz Biotechnology, Santa Cruz, CA, USA, SC-40, 1:250). Secondary antibodies were: goat anti-mouse IgG Alexa Fluor 680 (1:10,000) and goat anti-rabbit IRDye800 (Rockland, Philadelphia, PA, USA, 1:10,000). Protein bands were visualized using an infrared-based imaging system (Odyssey, LI-COR Biotechnology, Lincoln, NE, USA) and band intensities measured using the Li-Cor Odyssey software version 2.1.15. Virus particle release efficiency was calculated as (VLP signal /(VLP signal + cell lysate signal)). Quantification analyses plot the data mean with error bars signifying plus or minus 1 standard deviation (SD).

### 2.5. Fluorescence Microscopy

HeLa cells (ATCC CCL-2) grown on poly-l-lysine treated cover slips were transfected with pCMV-Gag-EGFP alone or together with pLLEXP1-hTsg101-myc using Roche X-tremeGene transfection reagent. After 48 h, cells were fixed in 4% formaldehyde (Thermo-Fisher, Waltham, MA, USA) and permeabilized in 0.1% Triton X-100/PBS. Tsg101 was detected in samples by indirect immunofluorescence using anti-myc Mab (Santa Cruz, CA, USA, SC-40, 1:100) and Texas Red tagged goat anti-mouse IgG (Molecular Probes, Eugene, OR, USA, 1:400). Nuclei were stained with NucBlue reagent (Thermo Fisher). Z-stack images were captured on an inverted fluorescence/differential-interference contrast (dic) Zeiss Axiovert 200M deconvolving fluorescence microscope operated by AxioVision Version 4.5 software (Zeiss, Jena, Germany). Deconvolution image processing used the constrained iterative method (Zeiss AxioVision software). Protein co-localization was assessed in cells by determination of Pearson’s coefficient of correlation of the entire cell using Image J software (National Institutes of Health).

### 2.6. Yeast Two-Hybrid Assay

*Saccharomyces cerevisiae* strain Y190 (genotype: ura3-52, his3-200, lys2-801, ade2-101, trp1-901, leu2-3, 112, gal4Δ, met-, gal80Δ, LYS2::GAL1UAS-HIS3TATA-HIS3, URA3::GAL1UAS-GAL1TATA-lacz) was used to test protein-protein interactions between HIV-1 Gag p6 or p6-Ub and Tsg101 wild-type or mutants using the Matchmaker GAL4 Yeast Two Hybrid beta-galactosidase assay (Clontech Laboratories, Inc., Mountain View, U.S.A.). pGAD plasmids and pGBT plasmids were co-transformed into Y190 and selected on minimal media plates lacking leucine and tryptophan. Transformants were inoculated intoselective liquid media (standard minimal media minus Leu, minus Trp), grown overnight at 30 °C, and then supplemented with an equal volume of yeast peptone-dextrose-media (YPD) to promote rapid growth for 4 h at 30 °C. Cell density was measured by reading OD650. Yeast cultures (1 mL) were washed in Z Buffer (60 mM Na_2_HPO_4_, 40 mM NaH_2_PO4, 10 mM KCl, 1 mM MgSO4) and resuspended in 0.5 mL reaction mix (Z buffer, 38 mM βME, 0.02% SDS, 0.02% Triton X100, 1 mg/mL ortho-nitrophenyl β-galactoside). Reactions (0.5 mL) were stopped by the addition of 0.25 mL of 1 M Na_2_CO_3_, cells pelleted, and the β-Gal signal read at OD415. A blank consisting of reaction mix was run in parallel and subtracted from the sample values. For the analysis of the Tsg101 mutants, two to three separate plasmid constructs were transformed and tested. Values were adjusted for cell density (OD650) and normalized to co-transformants of Tsg101 WT and p6 or p6-Ub run in parallel. Co-transformants of Tsg101 WT plus Gag p6 P7L (negative control) and Tsg101 WT plus Gag p6 2XPTAP (positive control) were run in parallel for each assay.

## 3. Results

### 3.1. The Tsg101 UEV Domain Binds tRNA

**Figure 1:**
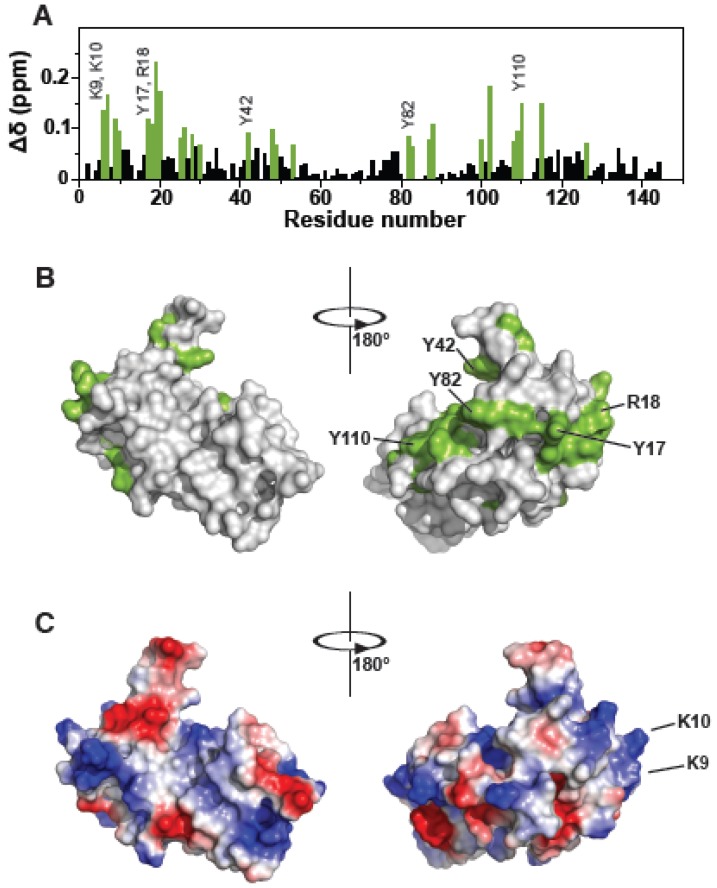
Yeast tRNA binds to Tsg101 UEV domain. (**A**), Residue-specific chemical shift perturbations of Tsg101 UEV upon binding to yeast tRNA (1:1). (**B**), Large perturbations (>1.5 s.d. from zero) are highlighted in green on a surface (grey) representation of the UEV (PDB ID: 1KPP; [42]). (**C**), Distribution of negative (red) and positive (blue) charges on a surface representation (grey) of the UEV domain (PDB ID: 1KPP; [42]).

The fact that (i), RNA is involved in the NC-Alix interface [1,39,40]; (ii), in the presence of RNA, Tsg101 is pulled-down with NC from cell lysates and in vitro [11,17] and (iii), small molecules that resemble nucleosides can bind the Tsg101 UEV domain [32,33] collectively suggested that Tsg101 might interact with RNA directly. To test this notion, we determined whether the UEV domain of Tsg101 recognized nucleic acid. As interactions with tRNA constitute the most frequent binding event between cytosolic Gag and RNA [41], a commercial mixture of yeast tRNA was tested. Using NMR spectroscopy, we mapped a tRNA binding site to a recombinant Tsg101 UEV protein on the face opposite to that containing the mono-Ub-, P(T/S)AP- and prazole-binding pockets [32,33,42]; Figure 1). Attempts using RNA Bind-n-Seq (RBNS) [43] to determine if UEV recognition was based on a specific subset of tRNA and to obtain a quantitative assessment of the binding provided no evidence for sequence-specific binding (Haque, Hogg, Strickland, and Tjandra, unpublished observations). 

**Figure 2:**
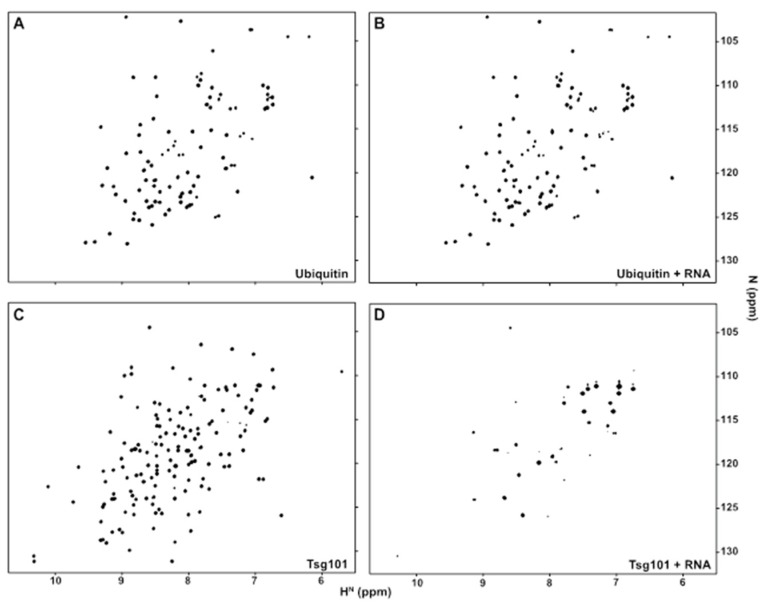
Yeast tRNA binds to Tsg101 UEV domain but not ubiquitin. ^1^H/^15^N-HSQC spectra of (**A**), free ^15^N-Ub^S57C^ or (**B**), ^15^N-Ub^S57C^ in the presence of yeast tRNA (1:1 ratio); (**C**), ^15^N-Tsg101 UEV domain; or (**D**), ^15^N-Tsg101 UEV domain:yeast tRNA complex.

Figure 1A shows the residues in the UEV perturbed by tRNA binding. Panels B and C show surface renderings of the Tsg101 UEV (PDB: 1KPP) with the tRNA chemical shift perturbations colored green (panel B) or with surface charges colored red (negative) or blue (positive) (panel C). Four Tyr residues, spaced in different patches (Y17, near the N-terminus; Y42, in the β-hairpin region; Y82 in the vestigial active site lip; and Y110, the highly conserved substitution for the catalytic Cys residue in the UEV vestigial active site) and four basic residues clustered near N-terminal helices 1 and 2 (K9, K10, K17, R18) exhibited large chemical shift perturbations (CSPs) when tRNA was added. As shown in panels B and C, the affected residues formed a dispersed patch on the face of the UEV opposite from the previously described mono-Ub- and P(T/S)AP-binding pocket [44,45,46] but otherwise did not closely follow the positive charge distribution on the UEV face, indicating no correlation between electrostatic charge and tRNA binding interface. Like mono-Ub and P(T/S)AP peptides, the distal domain of K63-linked di-Ub binds in the region of the β-hairpin (Strickland, submitted). The proximal domain of K63-di-Ub binds at the vestigial active site preferentially (Strickland, submitted). In contrast, tRNA binding determinants lay differentially in N-terminal helices-1 and -2. Based on the degree of chemical shift perturbance (CSP), tRNA is expected to bind significantly more strongly than Ub. We considered the possibility that tRNA might bind the UEV adventitiously. To check this, we determined by NMR whether tRNA binds Ub. As shown in Figure 2, tRNA bound the Tsg101 UEV but not Ub. Finding that the UEV recognized specific sequence and fold properties in the tRNA mixture that were not recognized in Ub makes it less likely that the RNA bound the UEV adventitiously.

### 3.2. tRNA Binding Does Not Prevent Access to the Site Necessary for Mono-Ub Binding

**Figure 3:**
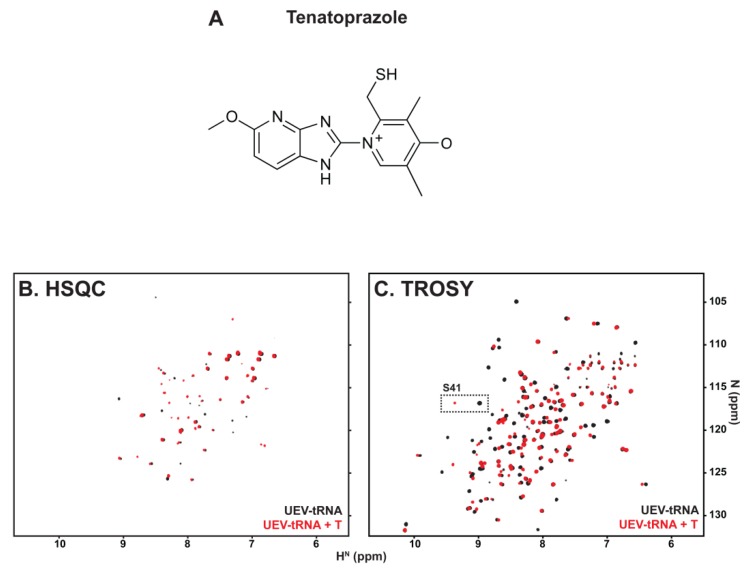
tRNA binding does not prevent access to sites necessary for Ub binding. (**A**), Chemical structure of active tenatoprazole (T) compound that targets Cys73 in the Tsg101 UEV domain [32]. (**B**), (**C**), Overlay of the HSQC spectra (**B**) or TROSY spectra (**C**) of the UEV-tRNA complex captured in the absence (black) or presence (red) of T.

To determine whether binding tRNA might interfere with Ub binding, we took advantage of the fact that small molecules in the prazole family (Figure 3, panel A) target the β-hairpin site [32,33], where mono-Ub and the distal domain of di-Ub bind ([42], Strickland, submitted), to test if pre-treatment with tRNA blocked prazole binding. Panel B shows an overlay of the heteronuclear single quantum coherence (HSQC) spectra of the UEV-tRNA complex captured in the absence (black) or presence (red) of tenatoprazole. Several of the signals produced by UEV-tRNA interaction were perturbed by tenatoprazole addition, indicating that binding tRNA did not prevent the interaction with the molecule. This finding was confirmed by transverse relaxation optimized spectroscopy (TROSY; panel C). The position of the signal assigned to residue S41 in the β-hairpin of the UEV-RNA complex was identified and found to undergo significant shift upon addition of tenatoprazole. The results indicate that tRNA binding did not prevent access to sites necessary for Ub binding.

### 3.3. The UEV Domain Is Not Required when Tsg101 Is at the Budding Site

**Figure 4:**
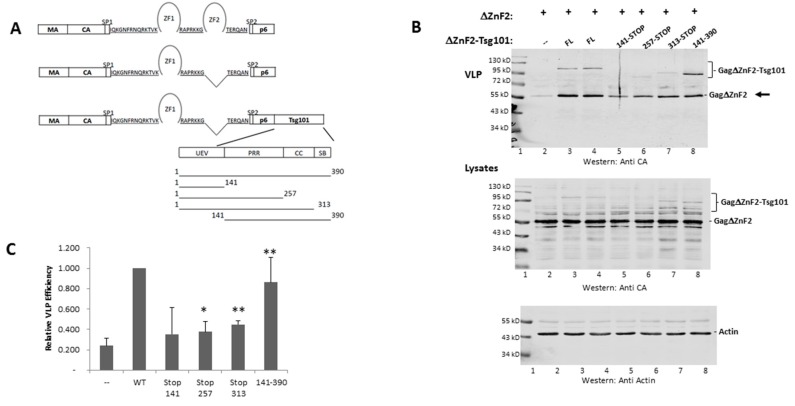
The UEV domain is not required when Tsg101 is at the budding site. (**A**), Schematic representation of Gag, Gag-ΔZnF_2_ and chimeric Gag-ΔZnF_2_-Tsg101 constructs used in this study. (**B**), Gag-ΔZnF_2_ was transfected alone or with chimeric Gag-ΔZnF_2_-Tsg101 and examined by Western analysis for release of the VLPs assembled from Gag-ΔZnF_2_ (see arrow). The cell lysate signal was normalized for total protein content using the cellular protein actin as an internal marker. (**C**), Quantitative analysis. Efficiency of VLP release was measured as (Gag-ΔZnF_2_ VLP/(Gag ΔZnF_2_ VLP + Gag-ΔZnF_2_ in lysate)) relative to Gag WT. As judged by Student’s *t*-test, asterisks (* *p* < 0.05; **p < 0.01) indicate the chimera which improved budding efficiency relative to Gag-ΔZnF_2_ transfected alone.

Chamontin et al. demonstrated that providing Tsg101 at the assembly site in the context of the chimeric Gag-ΔZnF_2_-Tsg101 construct was sufficient to rescue the release of a mutant lacking ZnF_2_ (Figure 4, panel A schematic), the element in NC required for Tsg101 recognition. Employing a chimeric protein to deliver Tsg101 to the assembly site circumvents technical limitations associated with “hyper”-expression of the Tsg101 protein arising from the tight cellular control of its steady-state level [47]. Their findings and the subsequent studies conducted by El Meshri et al. [15] showed that Tsg101 recruitment requires contributions from both the NC and p6 domains. We confirmed the finding that providing Tsg101 at the budding site by translational fusion of Tsg101 to Gag-ΔZnF_2_ (creating Gag-ΔZnF_2_-Tsg101) promoted release of the Gag-ΔZnF_2_ mutant (panel B, compare lane 2 to lanes 3 and 4). Quantitative analysis (panel C) indicated that providing WT Tsg101 in the context of the chimeric Gag-Tsg101 protein to the ΔZnF_2_ mutant promoted egress of the mutant about five-fold. The UEV domain alone (aa 1–140) or extended to include the coiled-coil region (i.e., to aa 313) rescued ΔZnF_2_ to a lesser but reproducible degree (panel B, compare lane one to lanes 5–7; panel C). In contrast, determinant(s) downstream of the UEV (aa 140–390) had significant impact and more closely approximated the full-length Tsg101 protein (panel B, lane 8; panel C). In all cases, the cell lysate signal was normalized for total protein content using the cellular protein actin as an internal marker (panel B, bottom). We conclude that the UEV region was not required when Tsg101 was provided at the budding site. This observation supports the conclusion that the NC domain contributes to Tsg101 recruitment, which is the function that is mediated through the UEV domain.

**Figure 5:**
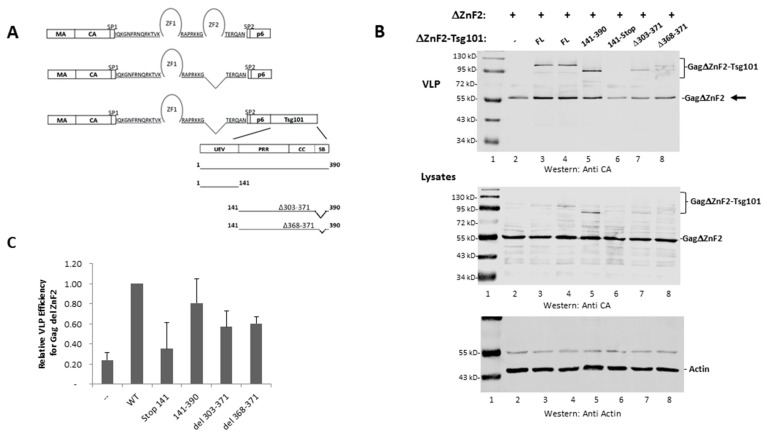
Vps28 binding is a critical event for ΔZnF_2_ rescue. (**A**), Schematic representation of Gag, Gag-ΔZnF_2,_ and chimeric Gag-ΔZnF_2_-Tsg101 constructs used in this study. (**B**), Gag-ΔZnF_2_ was transfected alone or with chimeric Gag-ΔZnF_2_-Tsg101 and examined by Western analysis for release of the VLPs assembled from Gag-ΔZnF_2_ (see arrow). The cell lysate signal was normalized for total protein content using the cellular protein actin as an internal marker. (**C**), Quantitative analysis. Efficiency of VLP release was measured as (Gag-ΔZnF_2_ VLP/(Gag ΔZnF_2_ VLP + Gag-ΔZnF_2_ in lysate)) relative to Gag WT. As judged by Student’s *t*-test, Tsg101 Δ303-371 and Δ368-371 differed from the chimera with Tsg101 sequences 141-390 (*p* < 0.05).

Previous studies demonstrated that the C-terminal helical domain of Tsg101, in particular residues 303-371, binds to Vps28 [48]. The core binding site lies within the tetrapeptide amino acids 368-371 [49]. The integrity of the Tsg101 C-terminal region is also important for binding of another ESCRT-I component, Vps37 [50]. To determine the contribution of determinants of ESCRT-I formation to rescue when ΔZnF_2_ was co-expressed with the chimeric Gag-Tsg101 protein, two deletion mutations, Δ303-371 and Δ368-371, were engineered within the full-length Tsg101 protein (Figure 5, panel A schematic). Deletion of the small region sufficed to disrupt rescue of ΔZnF_2_ egress to a significant degree (panel 5B), indicating that the missing residues were critical. Finding that this region is required for egress of ΔZnF_2_ suggests that recruitment of the ESCRT-I partners is part of the NC contribution to budding rather than a role fulfilled by Tsg101 recruited by the p6 domain. Possibly, the Vps28 binding site on Tsg101 is not accessible prior to Gag recruitment of Tsg101 to the cell periphery and/or its recruitment requires the Gag/Tsg101 multimerization that occurs at the assembly site.

### 3.4. Recruitment from the Cell Interior Requires the tRNA Binding Determinants

**Figure 6:**
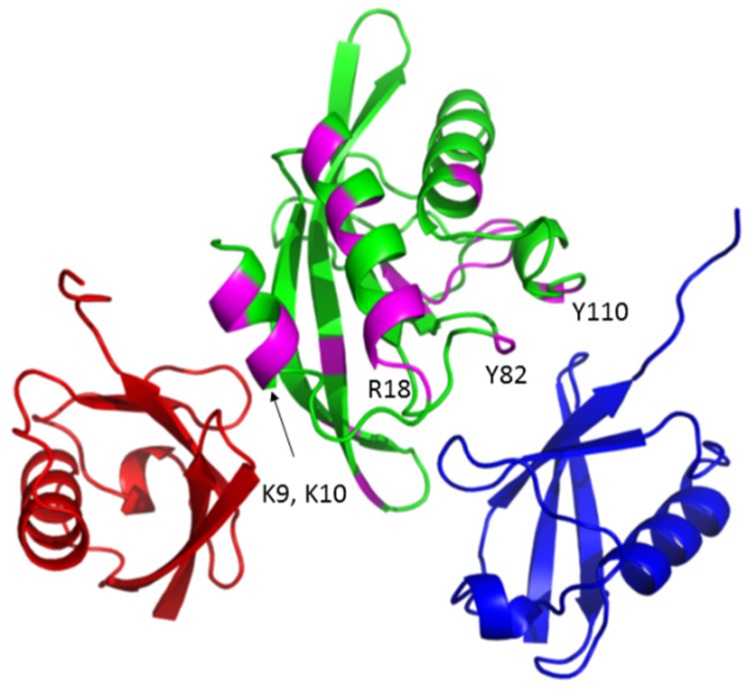
Proximity of tRNA and Ub-binding sites. The model shows the sites of determinants of tRNA binding on the UEV relative to the positions occupied by the proximal domain of K63-linked di-Ub (orange, N-terminal proximal; blue, vestigial active site vicinity). Determinants that recognize tRNA preferentially (located near α-helices-1 and -2) are colored magenta.

As noted above, the UEV domain of Tsg101 recognizes K63-linked di-Ub (Strickland, submitted). Figure 6 shows the major sites of tRNA CSPs (colored magenta) on the UEV (green) relative to the sites that can be occupied by the di-Ub proximal domain (orange and blue). Major determinants of tRNA binding were localized mainly in α-helices-1 and -2 (residues 6, 7, 9, 10, 17, 18, 19, 20); the major determinants of di-Ub binding lie in the vicinity of the vestigial active site (109, 111, 115, 119, 121, 122, 124, 126, 129, 138, 142).

**Figure 7:**
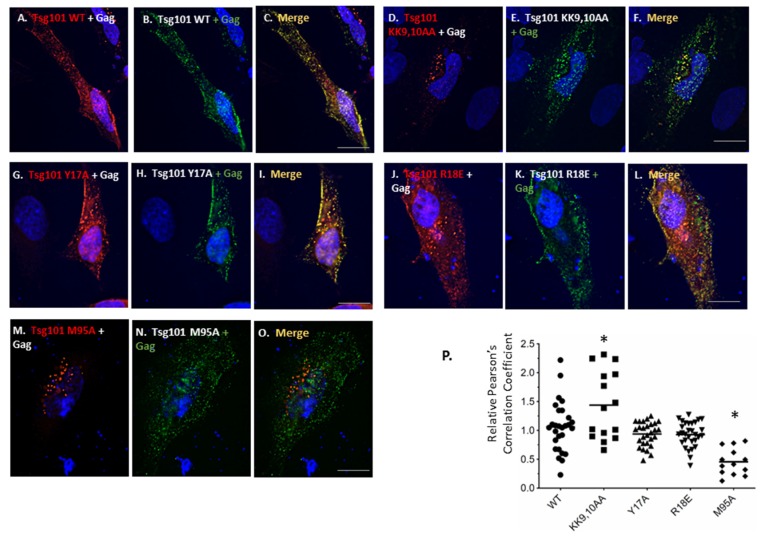
Recruitment from the cell interior requires tRNA-binding site. HIV-1 Gag-GFP was co-expressed with Tsg101 WT (panels **A**–**C**), Tsg101 KK9,10AA (panels **D**–**F**), Tsg101 Y17A (panels **G**–**I**), Tsg101 R18E (panels **J**–**L**), and Tsg101 M95A (panels **M**–**O**) and examined for the ability to co-localize. Images were captured on an inverted fluorescence/differential-interference contrast (dic) Zeiss Axiovert 200M fluorescence microscope. Scale bar equals 10 μm. Scatter plot of relative Pearson’s correlation coefficients assessment of co-localization is shown in panel **P**. Co-localization was determined for the entire co-transfected cell using NIH Image J, JACoP plugin software. Each Tsg101 mutant was scored relative to the average value for Tsg101 WT run in the same assay. By Student’s *t*-test analysis, KK9,10AA (*p* < 0.05) and M95A (*p* < 0.001) were significantly different from WT as indicated by the asterisks.

**Figure 8:**
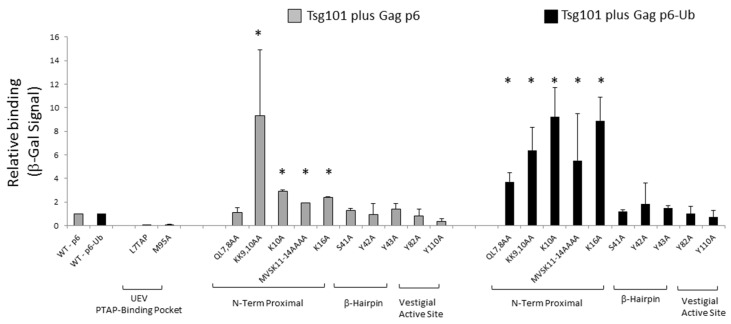
Mutation of tRNA binding sites permits recognition of Ub-modified Gag. The yeast 2-hybrid assay was used to measure the relative binding for p6 plus Tsg101 wild-type or mutants and p6-Ub plus Tsg101 wild-type or mutants. The figure shows the average for transformants representing two or three isolates for each mutation. The asterisks indicate the mutants that were different from Tsg101 WT by Student’s *t*-test (* *p* < 0.05). For each assay, wild-type controls (*n* = 3) were run in parallel with experimental samples and were used to normalize values. Controls also included Gag p6 L_7_TAP plus Tsg101 wild-type (negative control) and Gag p6 2xPTAP plus Tsg101 wild-type (positive control) for each assay.

To determine the impact of tRNA binding on the ability of Gag to recruit Tsg101 to the plasma membrane, a necessary step for virus budding [12,38], the residues that exhibited significant CSPs were mutated to Ala in Myc-tagged Tsg101. As noted above, the binding surface is extensive, but the residues exhibiting the largest CSPs were clustered near α-helices -1 and -2. The mutants were tested for impact on recruitment to the cell periphery following co-expression with Gag-GFP in HeLa cells. As shown in Figure 7, the double mutation KK9,10AA significantly increased co-localization of Tsg101 and Gag in the cell. Unlike the WT Tsg101 protein, however, there was no detectable co-localization at the plasma membrane (compare KK9,10AA, panels D,E,F to WT Tsg101, panels A,B,C). This indicates that these determinants impacted the signals that direct Gag to the plasma membrane, i.e., the tri-partite membrane-binding signal in the MA domain of Gag that directs myristate exposure, PI(4,5)P_2_ binding, and electrostatic interactions, including tRNA binding to MA ([51,52,53,54]; reviewed in [55]). This outcome was not exhibited by Y17A or R18E (panels G–L). As expected, Tsg101 M95A which removes Tsg101′s binding site for Gag p6, failed to co-localize with Gag on the plasma membrane or within the cell (panels M–O). The negative impact of the KK9,10AA mutations was not due to a global structural defect since the mutant retained the ability to co-localize with Gag (KK9,10,AA, panels D,E,F) and to interact with Gag p6 directly (see Figure 8 below). Control experiments confirmed that all of the mutants retained the ability to be recruited to the early endosome by Hrs (Strickland, submitted). Pearson’s correlation coefficients for assessment of co-localization are summarized (panel P).

### 3.5. Mutation of tRNA Binding Determinants Promotes Recognition of Ub-Modified Gag

Previous studies demonstrated that translational fusion of Ub to p6 increases its binding affinity to the UEV domain [42]. We therefore hypothesized that tRNA binding to the UEV might hinder access to Ub/di-Ub sites important for productive Tsg101-Gag interaction [32]. This hypothesis is supported by the observation that disruption of Gag-Tsg101 interaction through the PTAP motif results in accumulation of K48-linked Ub chains on the Gag protein [56]. Modification by K48-linked Ub chains is known to signal delivery of substrates to degradative compartments (reviewed in [57,58]). If tRNA-binding prevents recognition of, e.g., Ub-binding sites on Gag, then mutation of the tRNA binding determinants might promote Gag-Ub-Tsg101 interaction. To test the hypothesis, the yeast 2-hybrid assay was employed to assess the effect of mutations on tRNA binding determinants on Tsg101′s recognition of Gag p6. As shown in Figure 8, mutation of tRNA-binding determinants near helices 1 and 2 promoted p6-Tsg101 interaction particularly following translational fusion of Ub to the p6 C-terminus. We conclude that tRNA binding controls recognition of Ub-modified Gag and, thereby, Tsg101 recruitment and site of delivery. Thus, RNA- and Ub- binding to the Tsg101 UEV domain are critical to the mechanism by which Tsg101 facilitates Gag particle assembly and budding.

## 4. Discussion

Previous studies employing a cell line that expresses Gag/Gag-Pol and Rev constitutively revealed that co-immunoprecipitation of NCp15 from cell lysates by anti-Tsg101 antibodies was RNase-sensitive (VerPlank 2001, PhD Dissertation). Using lysates from cells expressing non-coding HIV-1 gRNA, GST-tagged NCp15, GST-tagged NCp9, GST-tagged NCp15ΔZnF_2_ or GST alone, Chamontin et al. [17] identified ZnF_2_ as the element in the protein critical for Tsg101 recognition. El Meshri et al. identified several residues in NC that were perturbed when Tsg101 bound the PTAP motif in Gag, suggesting that NC contributes to the Gag-Tsg101 interaction [15].The study reported herein provides direct evidence that Tsg101 can bind RNA through its UEV domain and demonstrates that the interaction controls Tsg101 recruitment. RNA-binding might facilitate Gag recruitment by reducing its interaction with endosomal membranes in a manner analogous to how tRNA interaction with MA is believed to minimize its sequestering on internal membranes [41,54]. Alternatively or in addition, UEV-RNA interaction with C-terminally located Lys residues, in conjunction with UEV P(T/S)AP-binding pocket engagement of Tsg101′s C-terminally located PSAP motif, might promote formation of an inactive conformation. Conceivably, this conformation could protect the Tsg101 protein from Ub ligases such as the Tsg101-associated ligase (TAL) that target C-terminally located Lys residues for polyubiquitination, ultimately leading to protein degradation [49,59]. We presume that any inactive Tsg101 conformation could be activated by partners like Gag, which possess a P(T/S)AP motif that is perhaps aided by Gag ubiquitination [42,60]. An interaction between the P(T/S)AP-binding pocket in the Tsg101 UEV domain and the PTAP motif in the protein’s C-terminal region could maintain the protein in a closed conformation until challenged by presentation of a PTAP motif in a context with greater binding affinity.

**Figure 9:**
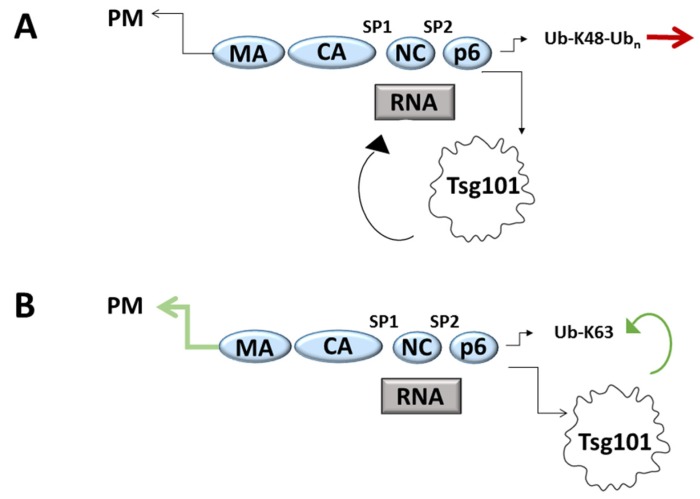
Model: tRNA binding controls Tsg101 recruitment and recruitment of Ub-modified Gag. Panel **A**, Tsg101 recruitment to the plasma membrane is suggested to be facilitated and stabilized by its ability to interact with the RNA bound to the NC domain in Gag. Under this condition, Lys residues in the C-terminal p6 region are not protected from possible non-productive Ub modification. Panel **B**, at the viral assembly site, Tsg101 with an available K63-di-Ub region may “cap” a Ub-modified Lys residue on p6 or another partner, sequestering it from further elongation or limiting its extension.

A model suggesting that RNA binding interferes with recognition of UEV Ub-binding sites important for productive Gag-Tsg101 interaction is proposed in Figure 9.

We hypothesize that UEV tRNA- and Ub- binding are linked activities that collectively regulate Gag-P(T/S)AP-Tsg101 interaction. Possibly, tRNA binding to the UEV facilitates and stabilizes Tsg101 recruitment by promoting interaction with NC-RNA. Any factor or event that disfavors the RNA-UEV interaction could permit the UEV Ub-binding site to interact with Ub molecules on Gag, Tsg101 itself or another ESCRT. Such “capping” might preclude chain growth beyond one or two molecules and, thereby, prevent non-productive targeting of the Tsg101-Ub-capped complex to the proteasome for degradation. Indeed, previous studies suggested the existence of two distinct types of E2/E3 substrates [61,62]. One type requires tRNA for conjugation of Ub; the other is tRNA independent. tRNA was shown to be involved in Ub marking of substrates rather than in degradation of Ub-protein conjugates. The pathways share common component(s) of the proteasome degradation system: the inhibition of one system accelerates the other. Insightfully, Ciechanover and collaborators had suggested that all protein conjugates are not destined for proteolysis, and that tRNA dependence might distinguish proteolytic versus nonproteolytic function(s) [61]. The UEV domain was not essential for ΔZnF_2_ rescue when Tsg101 was provided at the assembly site (c.f., Figure 4), however, determinants identified as differentially important for tRNA and di-Ub binding were critical for Tsg101 recruitment from the cell interior (c.f., Figure 7). Conceivably, the positioning of the di-Ub proximal domain at the N-terminus versus the vestigial active site could be tRNA-binding dependent. Supporting this notion, our studies in vitro indicate that the proximal domain of di-Ub dynamically exchanges between occupancy of the vestigial active site and the region that possesses the principal tRNA-binding determinants (i.e., the N-terminus) (Strickland, submitted). It will be of interest to determine in future studies whether N-terminal occupancy by RNA influences di-Ub binding and to identify the factors that contribute in the infected cell. In any case, this study has identified new targets for development of anti-viral agents and linked them to a critical event in viral replication.
